# Supplementary material for: Systematic assessment of structural variant annotation tools for genomic interpretation
Source: Life Sci Alliance. 2024 Dec 10;8(3):e202402949. doi: 10.26508/lsa.202402949 (PMC11632063; doi:10.26508/lsa.202402949)
Supplement: Supplementary file 1 [file LSA-2024-02949_TableS1.docx]

| **Supplementary Table S1. SV prioritization methods to be evaluated.** | | | | |
| --- | --- | --- | --- | --- |
|  | **Name of methods** | **Reference** | **Inclusion or not** | **Reason for exclusion** |
| 1 | SG-ADVISER CNV | Erikson et al. 2015 | No | Not available |
| 2 | VEP | McLaren et al. 2016 | No | Annotation only |
| 3 | SVScore | Ganel et al. 2017 | Yes | - |
| 4 | AnnotSV | Geoffroy et al. 2018 | Yes | - |
| 5 | ClinTAD | Spector and Wiita 2019 | No | Browser-based tool |
| 6 | TADfusion score | Huynh and Hormozdiari 2019 | No | Only for copy number deletion |
| 7 | ClassifyCNV | Gurbich and Ilinsky 2020 | Yes | - |
| 8 | SVFX | Kumar et al. 2020 | No | Failed during running |
| 9 | AutoCNV | Fan et al. 2021 | No | Manual review and adjustment are needed |
| 10 | ClinSV | Minoche et al. 2021 | No | Bam files are required |
| 11 | CNVxplorer | Requena et al. 2021 | No | Browser-based tool |
| 12 | nanotatoR | Bhattacharya et al. 2021 | No | Based on optical genome mapping |
| 13 | SVInterpreter | Fino et al. 2021 | No | Browser-based tool |
| 14 | CADD-SV | Kleinert and Kircher 2022 | Yes | - |
| 15 | DeepSVP | Althagafi et al. 2022 | No | Clinical phenotypes are required |
| 16 | strVctVre | Sharo et al. 2022 | Yes | - |
| 17 | SvAnna | Danis et al. 2022 | No | Based on long-read sequencing |
| 18 | svMIL | Nieboer and de Ridder 2020 | No | Somatic SVs only |
| 19 | SVPath | Yang et al. 2022 | No | Failed during running |
| 20 | TADA | Hertzberg et al. 2022 | Yes | - |
| 21 | TADeus2 | Poszewiecka et al. 2022 | No | Browser-based tool |
| 22 | X-CNV | Zhang et al. 2021 | Yes | - |
| 23 | CNV-ClinViewer | Macnee et al. 2023 | No | Browser-based tool |
| 24 | dbCNV | Lv et al. 2023 | Yes | - |
| 25 | phenoSV | Xu et al. 2023 | No | Clinical phenotypes are required |
| 26 | NeuroCNVscore | Liu et al. 2023 | No | Evaluation of neurodevelopmental SVs only |
| 27 | SCIP | Ding et al. 2023 | No | Bam files are required |
