# Supplementary material for: Systematic assessment of structural variant annotation tools for genomic interpretation
Source: Life Sci Alliance. 2024 Dec 10;8(3):e202402949. doi: 10.26508/lsa.202402949 (PMC11632063; doi:10.26508/lsa.202402949)
Supplement: Supplementary file 2 [file LSA-2024-02949_TableS2.docx]

| **Supplementary Table S2. Detailed description of seven independent data sources used in this study.** | | |
| --- | --- | --- |
| **Datasets** | **Description** | **Selection criteria** |
| GnomAD | gnomAD is a comprehensive database of exome and genome sequences from diverse populations. The gnomAD v4 SV dataset represents 63,046 genomes (GRCh38) (Collins et al. 2019). https://gnomad.broadinstitute.org/ | Negative set: SVs (deletion and duplication only) from gnomAD were selected with <1% population frequency (rare SVs), length-matched to the positive set, and no overlap with SVs in gnomAD V2 or positive sets. All gnomAD data were liftover to hg19. |
| ClinVar | ClinVar is a public archive of human genetic variations associated with disease, with supporting clinical evidence (access in March 2024) (Landrum et al. 2016). https://www.ncbi.nlm.nih.gov/clinvar/ | Positive set: SVs ≥ 50bp, marked as deletion, duplication, copy number loss and copy number gain, classified as ‘germline’, ‘pathogenic’, or ‘likely pathogenic’ with no conflicting evidence, and submitted between 2023-2024. |
|  |  | Negative set: ‘Benign’ or ‘likely benign’ germline SVs from ClinVar and submitted between 2023-2024; Additionally, rare SVs randomly selected from gnomAD v4 which are length-matched with positive SVs. |
| Noncoding SVs | Noncoding SVs are identified from peer-reviewed publications exploring their pathogenic effects (Gordon et al. 2014; Bieth et al. 2015; Turner et al. 2016; Cappuccio et al. 2019). | Positive set: Noncoding SVs (deletion and duplication only) from publications, excluding if any overlap with protein-coding genes (Gencode v30lift37).  Negative set: Randomly selected rare SVs from gnomAD v4, length-matched to the positive set, with no overlap with protein-coding genes (Gencode v30lift37). |
|  |  |  |
| Long range SVs | SVs implicated in long-range genome interactions, derived from peer-reviewed studies (Kouwenhoven et al. 2010; Ellaway et al. 2013; Tayebi et al. 2014; Lupianez et al. 2015; Franke et al. 2016; D'Haene et al. 2019; Long et al. 2020). | Positive set: published long-range SVs (deletion and duplication only) with no ≥70% reciprocal overlap within the dataset. |
|  |  | Negative set: rare SVs from gnomAD v4, length-matched to the positive set, with no overlap with known pathogenic SVs in ClinVar. |
| Somatic SVs | Somatic SVs from COSMIC v99, a curated database covering a variety of somatic mutations linked to cancer (Sondka et al. 2024) https://cancer.sanger.ac.uk/cosmic | Positive set: Somatic SVs (deletion and duplication only) from COSMIC (v99) with recurrence ≥ 2, located in high-risk genes listed in oncoKB. One record is excluded in the analysis on CADD-SV due to different reference genome assemblies. |
|  |  | Negative set: Randomly selected somatic SVs (deletion and duplication only) from COSMIC (v99) with recurrence = 1, with no overlap with high-risk genes in oncoKB. One record is excluded in the analysis on CADD-SV due to different reference genome assemblies. |
| Disease associated CNVs from a GWAS | Disease associated CNVs is derived from Auwerx et al. (Auwerx et al. 2024) , where population genetics were applied to UK Biobank exome sequencing to identify rare CNVs linked to quantitative traits and diseases. | Positive set: Rare, validated CNVs listed in the publication, supported by replication. Two records are excluded in the analysis on CADD-SV due to different reference genome assemblies. |
|  |  | Negative set: Rare SVs from gnomAD v4, length-matched to the positive set. Two records are excluded in the analysis on CADD-SV due to different reference genome assemblies. |
| Functional relevant SVs from eQTL studies | Functional relevant SVs are derived from Scott et al. ‘s study (Scott et al. 2021). a study mapping 61,668 SVs across 613 individuals from the GTEx project, showing their impact on gene expression and eQTLs. | Positive set: Rare SVs (deletion and duplication only) associated with aberrant gene expression across multiple tissues, with evidence of gene dosage changes. One record is excluded in the analysis on CADD-SV due to different reference genome assemblies.  Negative set: Rare SVs (deletion and duplication only) from gnomAD v4, length-matched to the positive set. One record is excluded in the analysis on CADD-SV due to different reference genome assemblies. |
|  |  |  |
