# Supplementary material for: Systematic assessment of structural variant annotation tools for genomic interpretation
Source: Life Sci Alliance. 2024 Dec 10;8(3):e202402949. doi: 10.26508/lsa.202402949 (PMC11632063; doi:10.26508/lsa.202402949)
Supplement: Supplementary file 3 [file LSA-2024-02949_TableS3.docx]

| **Supplementary Table S3. Performances over all approaches.** | | | | | | | | | | |
| --- | --- | --- | --- | --- | --- | --- | --- | --- | --- | --- |
| **Dataset** | **Software** | **Missing (%)** | **Accuracy** | **Sensitivity** | **Specificity** | **PPV** | **FPR** | **F1-Score** | **MCC** | **AUC** |
| Germline SVs | AnnotSV | 0 | 0.9 | 0.94 | 0.87 | 0.86 | 0.13 | 0.9 | 0.81 | 0.93 |
|  | CADD-SV | 0 | 0.84 | 0.79 | 0.91 | 0.93 | 0.09 | 0.85 | 0.69 | 0.9 |
|  | ClassifyCNV | 0 | 0.69 | 0.62 | 0.96 | 0.98 | 0.04 | 0.76 | 0.47 | 0.7 |
|  | dbCNV | 0 | 0.5 | 0.49 | 0.5 | 0.07 | 0.5 | 0.12 | 0 | 0.5 |
|  | StrVCTVRE | 0 | 0.94 | 0.91 | 0.97 | 0.97 | 0.03 | 0.94 | 0.88 | 0.96 |
|  | SVScore | 0 | 0.78 | 0.73 | 0.87 | 0.9 | 0.13 | 0.81 | 0.58 | 0.83 |
|  | TADA | 0 | 0.81 | 0.82 | 0.8 | 0.8 | 0.2 | 0.81 | 0.62 | 0.88 |
|  | XCNV | 0 | 0.9 | 0.94 | 0.87 | 0.86 | 0.13 | 0.9 | 0.81 | 0.91 |
| Noncoding SVs | AnnotSV | 0 | 0.83 | 1 | 0.75 | 0.67 | 0.25 | 0.8 | 0.71 | 0.83 |
|  | CADD-SV | 0.5 | - | - | - | - | - | - | - | - |
|  | ClassifyCNV | 0 | 0.58 | 1 | 0.55 | 0.17 | 0.45 | 0.29 | 0.3 | 0.58 |
|  | dbCNV | 0 | 0.58 | 1 | 0.55 | 0.17 | 0.45 | 0.29 | 0.3 | 0.58 |
|  | StrVCTVRE | 1 | - | - | - | - | - | - | - | - |
|  | SVScore | 0 | 0.92 | 1 | 0.86 | 0.83 | 0.14 | 0.91 | 0.85 | 0.86 |
|  | TADA | 0 | 0.92 | 1 | 0.86 | 0.83 | 0.14 | 0.91 | 0.85 | 0.92 |
|  | XCNV | 0 | 0.58 | 1 | 0.55 | 0.17 | 0.45 | 0.29 | 0.3 | 0.58 |
| Long range SVs | AnnotSV | 0 | 0.88 | 1 | 0.81 | 0.77 | 0.19 | 0.87 | 0.79 | 0.89 |
|  | CADD-SV | 0 | 0.88 | 1 | 0.81 | 0.77 | 0.19 | 0.87 | 0.79 | 0.89 |
|  | ClassifyCNV | 0 | 0.58 | 1 | 0.54 | 0.15 | 0.46 | 0.27 | 0.29 | 0.61 |
|  | dbCNV | 0 | 0.62 | 0.71 | 0.58 | 0.38 | 0.42 | 0.5 | 0.26 | 0.63 |
|  | StrVCTVRE | 0.12 | 0.91 | 0.92 | 0.91 | 0.92 | 0.09 | 0.92 | 0.83 | 0.89 |
|  | SVScore | 0 | 0.31 | 0.27 | 0.33 | 0.23 | 0.67 | 0.25 | -0.39 | 0.7 |
|  | TADA | 0 | 0.92 | 0.92 | 0.92 | 0.92 | 0.08 | 0.92 | 0.85 | 0.98 |
|  | XCNV | 0 | 0.96 | 1 | 0.93 | 0.92 | 0.07 | 0.96 | 0.93 | 0.95 |
| Somatic SVs | AnnotSV | 0 | 0.7 | 0.74 | 0.67 | 0.57 | 0.33 | 0.64 | 0.4 | 0.74 |
|  | CADD-SV | 0.01 | 0.59 | 0.57 | 0.61 | 0.59 | 0.39 | 0.58 | 0.19 | 0.59 |
|  | ClassifyCNV | 0 | 0.69 | 0.85 | 0.64 | 0.43 | 0.36 | 0.57 | 0.42 | 0.7 |
|  | dbCNV | 0 | 0.52 | 0.47 | 0.52 | 0.04 | 0.48 | 0.08 | 0 | 0.5 |
|  | StrVCTVRE | 0.25 | 0.62 | 0.69 | 0.53 | 0.63 | 0.47 | 0.66 | 0.22 | 0.65 |
|  | SVScore | 0 | 0.52 | 0.5 | 0.55 | 0.56 | 0.45 | 0.53 | 0.05 | 0.54 |
|  | TADA | 0.36 | 0.72 | 0.38 | 0.92 | 0.73 | 0.08 | 0.5 | 0.37 | 0.77 |
|  | XCNV | 0 | 0.64 | 0.6 | 0.72 | 0.78 | 0.28 | 0.68 | 0.31 | 0.68 |
| SVs from a GWAS | AnnotSV | 0 | 0.67 | 0.76 | 0.63 | 0.5 | 0.37 | 0.6 | 0.37 | 0.71 |
|  | CADD-SV | 0.06 | 0.68 | 0.82 | 0.63 | 0.47 | 0.37 | 0.6 | 0.41 | 0.68 |
|  | ClassifyCNV | 0 | 0.61 | 0.77 | 0.57 | 0.31 | 0.43 | 0.44 | 0.27 | 0.63 |
|  | dbCNV | 0 | 0.53 | 0.57 | 0.52 | 0.25 | 0.48 | 0.35 | 0.08 | 0.54 |
|  | StrVCTVRE | 0.22 | 0.68 | 0.66 | 0.72 | 0.81 | 0.28 | 0.72 | 0.36 | 0.67 |
|  | SVScore | 0 | 0.38 | 0.38 | 0.37 | 0.41 | 0.63 | 0.39 | -0.25 | 0.57 |
|  | TADA | 0.02 | 0.65 | 0.64 | 0.67 | 0.68 | 0.33 | 0.66 | 0.3 | 0.6 |
|  | XCNV | 0 | 0.69 | 0.68 | 0.7 | 0.72 | 0.3 | 0.7 | 0.38 | 0.72 |
| Functional relevant SVs from an eQTL study | AnnotSV | 0 | 0.64 | 0.58 | 0.95 | 0.99 | 0.05 | 0.73 | 0.39 | 0.64 |
|  | CADD-SV | 0.01 | 0.65 | 0.64 | 0.67 | 0.7 | 0.33 | 0.67 | 0.31 | 0.68 |
|  | ClassifyCNV | 0 | 0.6 | 0.55 | 1 | 1 | 0 | 0.71 | 0.33 | 0.58 |
|  | dbCNV | 0 | 0.5 | 0.5 | 0.5 | 0.08 | 0.5 | 0.14 | 0 | 0.5 |
|  | StrVCTVRE | 0.32 | 0.4 | 0.59 | 0.27 | 0.35 | 0.73 | 0.44 | -0.15 | 0.53 |
|  | SVScore | 0 | 0.53 | 0.53 | 0.54 | 0.57 | 0.46 | 0.55 | 0.07 | 0.54 |
|  | TADA | 0 | 0.66 | 0.63 | 0.71 | 0.78 | 0.29 | 0.7 | 0.33 | 0.66 |
|  | XCNV | 0 | 0.69 | 0.66 | 0.75 | 0.81 | 0.25 | 0.73 | 0.4 | 0.71 |

- represents NA, no value available.
