# Supplementary material for: Systematic assessment of structural variant annotation tools for genomic interpretation
Source: Life Sci Alliance. 2024 Dec 10;8(3):e202402949. doi: 10.26508/lsa.202402949 (PMC11632063; doi:10.26508/lsa.202402949)
Supplement: Supplementary file 4 [file LSA-2024-02949_TableS4.docx]

| **Supplementary Table S4. Performance across approaches in two SV types.** | | |
| --- | --- | --- |
| **SV type** | **Software** | **AUC** |
| Deletion | AnnotSV | 0.93 |
|  | CADD-SV | 0.9 |
|  | ClassifyCNV | 0.71 |
|  | dbCNV | 0.5 |
|  | StrVCTVRE | 0.96 |
|  | SVScore | 0.85 |
|  | TADA | 0.89 |
|  | XCNV | 0.93 |
| Duplication | AnnotSV | 0.93 |
|  | CADD-SV | 0.94 |
|  | ClassifyCNV | 0.63 |
|  | dbCNV | 0 |
|  | StrVCTVRE | 0.95 |
|  | SVScore | 0.49 |
|  | TADA | 0.64 |
|  | XCNV | 0.91 |
