# Supplementary material for: Systematic assessment of structural variant annotation tools for genomic interpretation
Source: Life Sci Alliance. 2024 Dec 10;8(3):e202402949. doi: 10.26508/lsa.202402949 (PMC11632063; doi:10.26508/lsa.202402949)
Supplement: Supplementary file 5 [file LSA-2024-02949_TableS5.docx]

| **Supplementary Table S5. Performance across approaches in different length groups.** | | | | |
| --- | --- | --- | --- | --- |
| **SV type** | **Software** | **length<6*10^~3^** | **length=6*10~3-10^~5^** | **length>10^~5^** |
| Deletion | AnnotSV | 0.92 | 0.97 | 0.95 |
|  | CADD-SV | 0.92 | 0.81 | 0.9 |
|  | ClassifyCNV | 0.72 | 0.6 | 0.7 |
|  | dbCNV | 0.5 | 0.5 | 0.5 |
|  | StrVCTVRE | 0.97 | 0.96 | 0.95 |
|  | SVScore | 0.95 | 0.92 | 0.65 |
|  | TADA | 0.88 | 0.91 | 0.92 |
|  | XCNV | 0.94 | 0.93 | 0.93 |
| Duplication | AnnotSV | 1 | 0.8 | 0.94 |
|  | CADD-SV | 1 | 1 | 0.9 |
|  | ClassifyCNV | 0.79 | 0.8 | 0.53 |
|  | dbCNV | 0 | 0 | 0 |
|  | StrVCTVRE | 1 | 0.92 | 0.92 |
|  | SVScore | 0.69 | 0.56 | 0.47 |
|  | TADA | 0.98 | 0.6 | 0.54 |
|  | XCNV | 1 | 0.94 | 0.85 |
