# Supplementary material for: Systematic assessment of structural variant annotation tools for genomic interpretation
Source: Life Sci Alliance. 2024 Dec 10;8(3):e202402949. doi: 10.26508/lsa.202402949 (PMC11632063; doi:10.26508/lsa.202402949)
Supplement: Supplementary file 6 [file LSA-2024-02949_TableS6.docx]

| **Supplementary Table S6. Performance across approaches in different groups of genes.** | | | | | |
| --- | --- | --- | --- | --- | --- |
| **SV type** | **Software** | **Disease gene = 0** | **Disease gene > 0** | **Functional gene = 0** | **Functional gene > 0** |
| Deletion | AnnotSV | 0.92 | 0.84 | 0.94 | 0.9 |
|  | CADD-SV | 0.89 | 0.88 | 0.89 | 0.89 |
|  | ClassifyCNV | 0.81 | 0.55 | 0.74 | 0.55 |
|  | dbCNV | 0.48 | 0.51 | 0.49 | 0.51 |
|  | StrVCTVRE | 0.98 | 0.92 | 0.97 | 0.93 |
|  | SVScore | 0.85 | 0.87 | 0.82 | 0.9 |
|  | TADA | 0.94 | 0.75 | 0.93 | 0.75 |
|  | XCNV | 0.91 | 0.88 | 0.93 | 0.89 |
| Duplication | AnnotSV | 0 | 0.76 | 0.93 | 0.95 |
|  | CADD-SV | 0 | 0.85 | 0.93 | 1 |
|  | ClassifyCNV | 0 | 0.5 | 0.64 | 0.5 |
|  | dbCNV | 0 | 0.5 | 0.5 | 0.5 |
|  | StrVCTVRE | 0 | 0.95 | 0.88 | 1 |
|  | SVScore | 0 | 0.58 | 0.59 | 0.39 |
|  | TADA | 0 | 0.57 | 0.61 | 0.57 |
|  | XCNV | 0 | 0.86 | 0.83 | 1 |
