# Supplementary material for: Systematic assessment of structural variant annotation tools for genomic interpretation
Source: Life Sci Alliance. 2024 Dec 10;8(3):e202402949. doi: 10.26508/lsa.202402949 (PMC11632063; doi:10.26508/lsa.202402949)
Supplement: Supplementary file 7 [file LSA-2024-02949_TableS7.docx]

| **Supplementary Table S7. Curated datasets from publications for biological mechanisms.** | | | | | | |
| --- | --- | --- | --- | --- | --- | --- |
| **Type** | **Chromosome** | **Start** | **End** | **SV Type** | **Phenotype** | **Reference** |
| Noncoding SV | 5 | 14015350 | 14055499 | Duplication | Autism spectrum disorder | (Turner et al. 2016) |
|  | 11 | 131345836 | 131583798 | Deletion | Autism spectrum disorder | (Turner et al. 2016) |
|  | 16 | 6908075 | 7079700 | Deletion | Autism spectrum disorder | (Turner et al. 2016) |
|  | 14 | 43545282 | 43837068 | Deletion | Autism spectrum disorder | (Cappuccio et al. 2019) |
|  | 15 | 25257218 | 25375375 | Deletion | Prader–Willi syndrome | (Bieth et al. 2015) |
|  | 17 | 68680882 | 68965323 | Deletion | Pierre Robin sequence Cooks syndrome, sex reversal | (Gordon et al. 2014) |
| Long range SV | 2 | 221278232 | 223014332 | Deletion | Limb malfunction | (Lupianez et al. 2015) |
|  | 2 | 219907598 | 220954793 | Duplication | Limb malfunction | (Lupianez et al. 2015) |
|  | 5 | 88412388 | 91996699 | Deletion | Severe ID, seizures and hypotonia | (D'Haene et al. 2019) |
|  | 5 | 88232587 | 93437723 | Deletion | Developmental delay, seizures and hypotonia | (D'Haene et al. 2019) |
|  | 7 | 95624825 | 96135521 | Deletion | Split hand foot malformation, Hearing loss | (Tayebi et al. 2014) |
|  | 7 | 95667046 | 95872044 | Deletion | Split hand foot malformation | (Tayebi et al. 2014) |
|  | 7 | 95693341 | 95862369 | Deletion | Split hand foot malformation | (Tayebi et al. 2014) |
|  | 7 | 95615187 | 95783313 | Deletion | Split hand foot malformation | (Tayebi et al. 2014) |
|  | 7 | 95552064 | 96432064 | Deletion | Limb malfunction | (Kouwenhoven et al. 2010) |
|  | 14 | 29695258 | 30872374 | Deletion | Neurodevelopmental disease | (Ellaway et al. 2013) |
|  | 17 | 68020547 | 70038208 | Duplication | Cooks syndromes | (Franke et al. 2016) |
|  | 17 | 68663405 | 68738405 | Deletion | Pierre Robin sequence | (Long et al. 2020) |
